# Sex differences in postprandial blood glucose and body surface temperature are contingent on flight in the fruit bat, *Cynopterus sphinx*

**DOI:** 10.1242/bio.053926

**Published:** 2021-02-17

**Authors:** Venkatesh Nagarajan-Radha, Paramanantha Swami Doss Devaraj

**Affiliations:** 1 School of Biological Sciences, Monash University, Clayton Campus, Victoria 3800, Australia; 2 Centre for Behavioural and Immuno Ecology, Department of Zoology, St. John's College, Palayamkottai 627002, Tamil Nadu, India

**Keywords:** High-sugar diet, Hyperglycaemia, Intralocus sexual conflict, Nutritional ecology, Physical activity, Thermal imaging, Thermogenesis

## Abstract

The postprandial blood glucose level is very high for the body size in frugivorous bats. Like other homeotherms, bats release heat during digestion of dietary macronutrients. Despite males and females of the same species exhibiting different foraging behaviour, empirical support for sex differences in blood glucose and body surface temperature in fruit bats is poor. Moreover, while flight affects postprandial metabolism, whether such effects are different in each sex of fruit bats is unclear. Here, we studied these questions in the fruit bat, *Cynopterus sphinx*. We first assessed whether there are sex differences in the postprandial level of blood glucose and body surface temperature over time in rested bats. We then assessed whether flight affects outcomes of sex differences in both traits. We found that the estimated marginal means of both traits were generally higher in females than males, in rested bats. Notably, the sex difference in both traits was only significant at specific sampling time of the assay. Further, the trait means significantly differed between the sexes only in the rested, but not active, bats, meaning that signals of sex difference in metabolic traits eroded when bats were active. Taken together, our findings suggest that in *C. sphinx*, the sex specificity in the expression of metabolic traits is significantly dependent on physical activity.

## Abstract (precis)

**Summary:** The level of sex differences in metabolic traits is affected by flight in *Cynopterus sphinx*, a finding that has general implications for sex-specific life-history evolution in fruit bats.

## INTRODUCTION

Studying sex differences in trait expression is essential to understanding the evolution of animal life histories. The evolutionary theory of intralocus genetic conflict predicts that selection for optimal expression of a trait in one sex can facilitate selection for different magnitude of expression of the same trait in the other sex (Bonduriansky and Chenoweth, 2009; van Doorn, 2009). Sex-specific trait expression is underpinned by alleles that are commonly expressed, but are differently optimised for performance in each sex. It is therefore generally predicted that traits affecting individual fitness are optimised differently in each sex. Examples of traits that are differently expressed in each sex include longevity and metabolic rate in insects (Clancy, 2008; Nagarajan-Radha et al., 2020); and longevity and metabolism in mammals (Austad and Fischer, 2016; Clutton-Brock and Isvaran, 2007; Mauvais-Jarvis, 2015).

Metabolism provides the energy required for the expression of life-history traits. The pattern of allocation of available metabolic energy to different life-history traits is different between the sexes in animals (Tarka et al., 2018). Genes involved in metabolic pathways confer sex-specific life-history expression in mice and humans (Norheim et al., 2019; Partridge et al., 2005; Tower, 2017). Moreover, in general, phenotype expression in mammals is sex specific (Karp et al., 2017). Yet, sex difference in the expression of traits related to metabolism and life history is poorly studied in certain groups of animals, such as bats. Bats are a particularly interesting group to study metabolic traits because they are the only mammals that have evolved powered flight. Empirical studies have uncovered adaptations necessary for energy-intensive flight in bats. For instance, bats have a high rate of metabolism (i.e. quickly converting dietary macronutrients into energy), to support physical activity, compared to other similar-sized animals (Harrison and Roberts, 2000; Munshi-South and Wilkinson, 2010; Winter and von Helversen, 1998). Adaptation to flight has not only affected their metabolism but also their heart rate, respiration, immunity and longevity in bats (Ahn et al., 2019; Munshi-South and Wilkinson, 2010; O'Mara et al., 2017; Voigt and Winter, 1999).

Studying the relation between metabolism and flight is particularly intriguing in frugivore and nectarivore bats. Because, the postprandial blood glucose level is higher for their body size in these groups of bats (Amitai et al., 2010; Voigt and Speakman, 2007; Welch et al., 2008). The postprandial blood glucose level measured in bats kept at rest during assays is higher than 10 mmol/l (Kelm et al., 2011; Meng et al., 2016; Peng et al., 2017). Such high postprandial blood glucose (>10 mmol/l) confers hyperglycaemia in other similar-sized terrestrial mammals and is linked to ischemic stroke in humans (Bruno et al., 1999; Ceriello, 2003; Kelm et al., 2011; Umminger, 1975). But it is less clear whether fruit and nectar bats suffer from deleterious effects of hyperglycaemia.

Two findings are particularly noteworthy regarding frugivorous and nectarivorous bats’ proximate adaptations to sustain life in high-sugar diets: (1) heightened expression of digestive enzymes in the gut (Hernandez and Martinez del Rio, 1992; Ogunbiyi and Okon, 1976); and (2) their energy-intensive flight (Amitai et al., 2010; Kelm et al., 2011; Peng et al., 2017; Voigt and Speakman, 2007). Empirical studies demonstrate that flight significantly affects the level of postprandial blood glucose in fruit and nectar bats; demonstrating that these groups of bats have adapted to directly convert all exogenous sugar derived from their diet into fuel for flight (Amitai et al., 2010; Voigt and Speakman, 2007). Notwithstanding, homeothermic animals release heat during digestion of dietary macronutrients (Stock, 1999). Studies on lab models, such as mice and rats, show that the body surface temperature changes with the metabolic state of the animal (Huang et al., 2013; Nagashima et al., 2003). But researchers rarely investigate body surface temperature variation associated with the metabolic state in bats. Exceptions being studies on skin temperature changes during the inactive state of bats, such as torpor (Turbill et al., 2003). More generally, empirical support for sex-differences in metabolic trait expression in fruit and nectar bats is generally poor. Previous studies that have assayed for postprandial blood glucose in species of fruit and nectar-eating bats within a controlled environment have either used one sex in their experiment or pooled data from both sexes in their analyses and thus have not reported sex differences (Amitai et al., 2010; Kelm et al., 2011; Meng et al., 2016; Peng et al., 2017; Thomas, 1975; Voigt and Speakman, 2007).

The fruit bat, *Cynopterus sphinx,* is commonly found throughout Asia, from Pakistan in the West to Indonesia in the East (Storz and Kunz, 1999). Foraging ecology of each sex is different in *C. sphinx*, where male bats generally forage closer to their roosting sites than females (Marimuthu et al., 1998). Given that the foraging ecology differs between the sexes in these fruit bats, we hypothesised that the metabolic traits that underpin physical activity would be expressed differently in each sex. To test the hypothesis, we investigated sex differences in the postprandial levels of blood glucose and body surface temperature in adult non-reproductive bats. We designed four separate assays to evaluate (1) whether postprandial levels of blood glucose and body surface temperature are different between the sexes over time when fed bats are kept at complete rest until sampling each trait, and (2) whether sex-differences in each of the two traits are affected by flight. We predicted that female bats would exhibit higher trait means compared to males and that the level of sex difference in both traits would be affected by flight.

## RESULTS

### Metabolic traits are significantly different between the sexes in resting bats at the specific sampling time of the assays

**Fig. 1.**
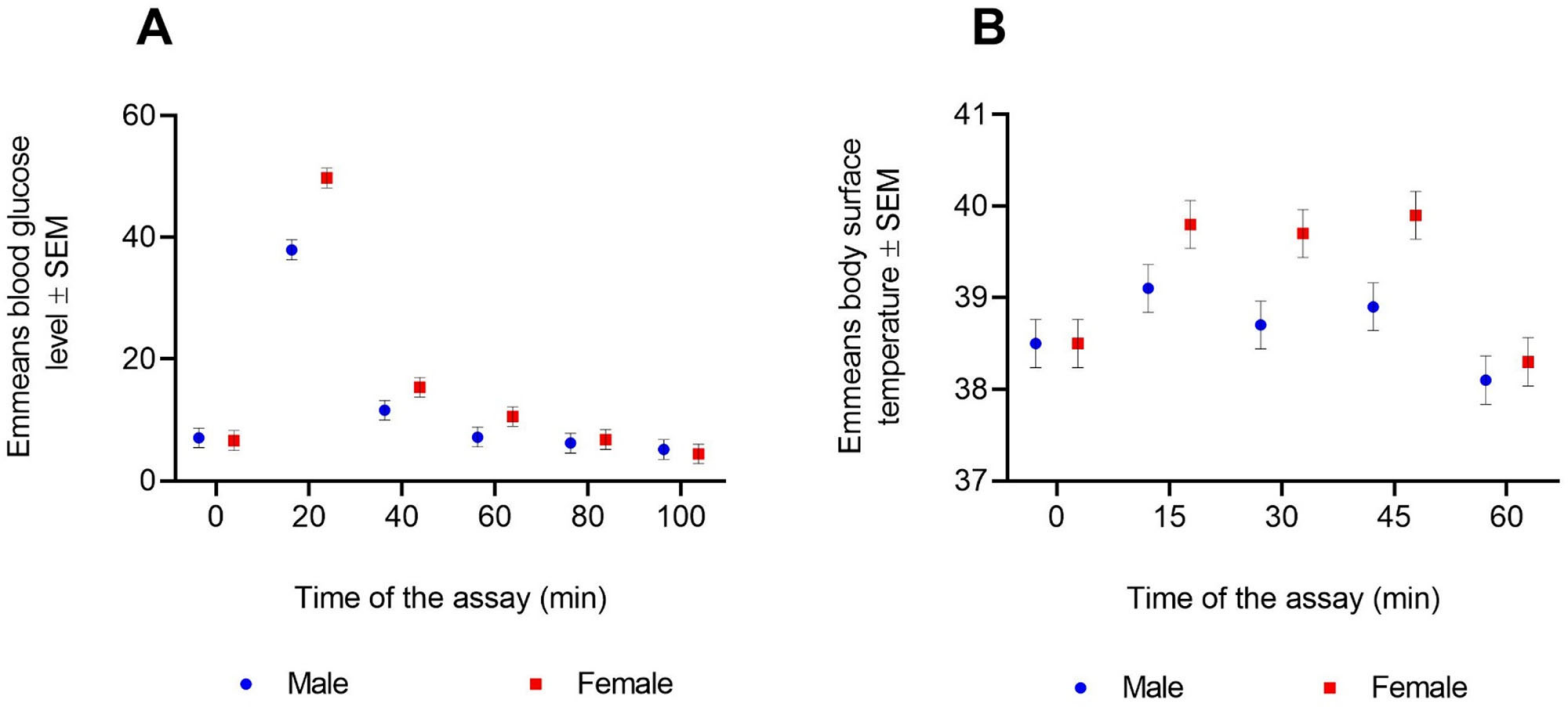
**Sex-differences in metabolic traits.** Sex differences in (A) emmeans postprandial blood glucose sampled across 0 to 100 min (*n*=4 bats per sex) and (B) emmeans postprandial body surface temperature sampled across 0 to 60 min (*n*=5 bats per sex). Each assay employed different individuals. The individuals were repeatedly sampled in each assay. 0 min indicates preprandial measurements, after which the individuals were each fed with 1.5 ml of standard diet and deployed for postprandial measurements made at specific sampling times (every 20 min in A and 15 min in B). The lmer models analysing data from these assays included body size index (ratio of body mass and forearm length) as a covariate and individual ID as a random effect. Emmeans (±s.e.m) for all pairwise combinations between sex and time of the assay were extracted from the lmer models. Emmeans allowed us to correct for effects attributable to variation in body size and repeated sampling from individuals. We analysed the models for significant difference in trait means across all pairwise combinations of sex and sampling times, within a post-hoc analytical framework using the TukeyHSD test in R. The postprandial blood glucose level was only significantly different between the sexes at 20 min (difference=11.65, *P*=0.0004). The body surface temperature of the sexes differed significantly at 30 min (difference=1.24, *P*=0.014) and 45 min (difference=1.26, *P*=0.012).

The time of the assay (*dredge* analysis: RVI=1) had a significant effect on the blood glucose level (Type III ANOVA: *P*<0.0001, Table 1A). The postprandial blood glucose level at 20 min was significantly different from the preprandial level. This level of increase was notably different in each sex: the difference was higher in females (Tukey HSD test: difference=43.13, *P*<0.0001) compared to male bats (difference=30.86, *P*<0.0001). The interaction term sex×time of the assay (RVI=0.006) had a significant effect on blood glucose level (Type III ANOVA: *P=*0.0268, Table 1A and Fig. 1A). Notably, the postprandial blood glucose level was only significantly different between the sexes at the 20 min sampling time of the assay (Tukey HSD test: difference=11.65, *P*=0.0004). The change in emmeans blood glucose level over time in each sex was similar to the pattern observed in the raw mean values (Fig. S1).

**Table 1.**
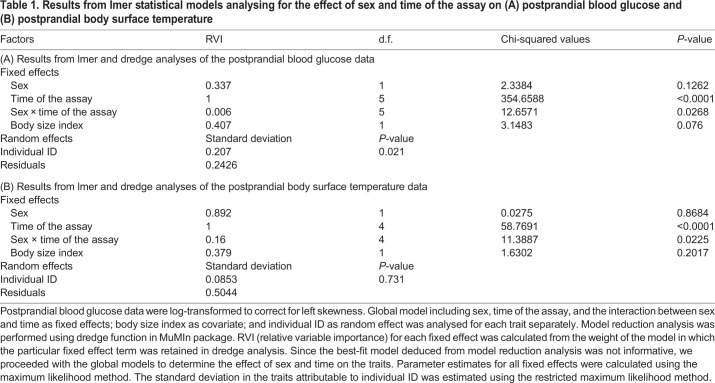
**Results from lmer statistical models analysing for the effect of sex and time of the assay on (A) postprandial blood glucose and (B) postprandial body surface temperature**

The predictor variable time (RVI=1) had a significant effect on body surface temperature (Type III ANOVA: *P*<0.0001, Table 1B). The postprandial level of body surface temperature measured at 15 min was significantly different from the preprandial level only in females (Tukey HSD test: difference=1.34, *P=*0.006) and not in males (difference=0.56, *P=*0.76). The interaction term sex×time of the assay (RVI=0.16) had a significant effect on the body surface temperature (Type III ANOVA: *P=*0.0225, Table 1B and Fig. 1B). The level of difference in body surface temperature between the sexes was significant at the 30 min (Tukey HSD test: difference=1.24, *P*=0.014) and 45 min sampling time of the assay (difference=1.26, *P=*0.012). The level of variation in emmeans body surface temperature over time in each sex was similar to the pattern observed in the raw means data (Fig. S2).

### Sex differences in metabolic traits are affected by flight

**Fig. 2.**
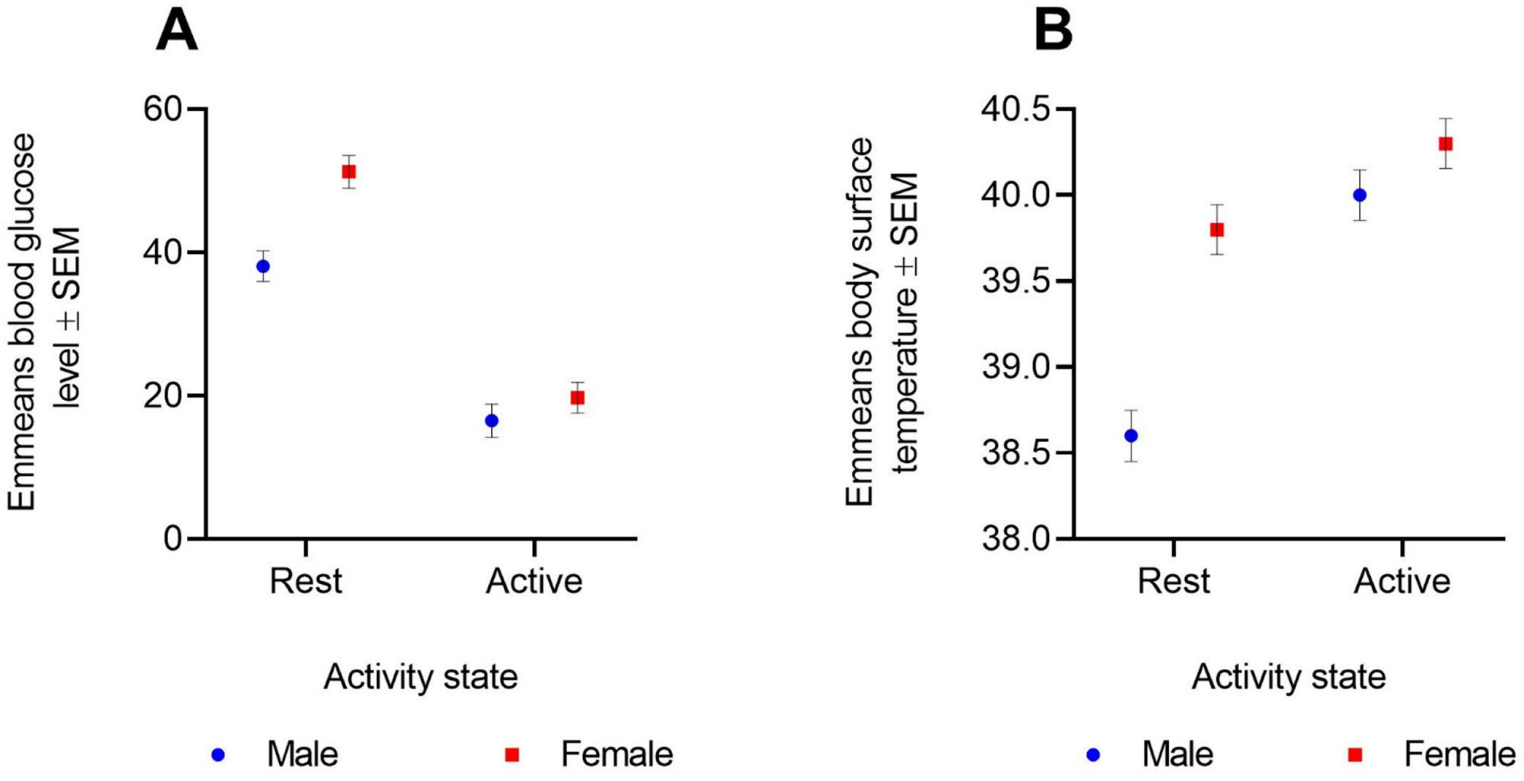
**Effect of flight on sex-differences in metabolic traits.** Effect of flight on outcomes of (A) postprandial blood glucose (*n*=4 bats per sex) and (B) postprandial body surface temperature (*n*=5 bats per sex). Different individuals were used in the rest and active groups in each assay. Individuals were sampled only once in each assay. In the rest group, each individual was fed with 1.5 ml standard diet and held in a cloth bag for 30 min before sampling the traits. In the active group, fed individuals were allowed to fly for 5 min before sampling the traits. The linear models included body size as a covariate, thus correcting the trait means for effects attributed to variation in body size of the individuals. Emmeans (±s.e.m) for all pairwise combinations of sex and activity-states were extracted from the linear models. TukeyHSD test was used to determine the significant differences in trait emmeans across all pairwise combinations of sex and activity-states. The postprandial blood glucose level differed significantly between the sexes only in the rest (Tukey HSD test: difference in emmeans=13.22, *P*=0.013), and not in the active group (Tukey HSD test: difference in emmeans=3.22, *P*=0.95). It was also the case in terms of postprandial body surface temperature: the trait means differed significantly between the sexes only in the rest (Tukey HSD test: difference in emmeans=1.2, *P*=0.0001), and not in the active group (Tukey HSD test: difference in emmeans=0.3, *P*=0.642).

Flight conferred a significant effect on the postprandial blood glucose level (ANOVA: *F*=142.3984, *P*<0.0001, Table 2A). The postprandial blood glucose level measured from individuals of each sex in the rest group was higher than in individuals in the active group (Fig. 2A). In males, the difference in emmeans postprandial blood glucose level between the two groups was 21.56 (Tukey HSD test: *P*<0.0005) and the difference was 31.56 in females (*P*<0.0001). The interaction term sex×activity-state had a significant effect on the blood glucose level (*F*=5.5094, *P=*0.0387, Table 2A and Fig. 2A). When comparing emmeans postprandial blood glucose between the sexes, we found the values to be significantly different only in the rested group (Tukey HSD test: difference in emmeans=13.22, *P=*0.013) and not in the active group (difference in emmeans=3.22, *P=*0.95).

**Table 2.**
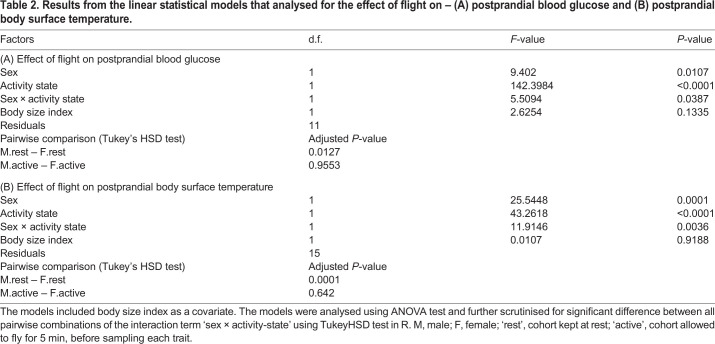
**Results from the linear statistical models that analysed for the effect of flight on – (A) postprandial blood glucose and (B) postprandial body surface temperature.**

Flight had a significant effect on the postprandial body surface temperature (*F*=43.2618, *P*<0.0001, Table 2B). Flight affected body surface temperature differently in each sex. The difference in body surface temperature among the two cohorts (active versus rested) was only significant in males (Tukey HSD test: difference in emmeans=1.437, *P*<0.0001) and not in females (difference in emmeans=0.463, *P*=0.15). The interaction term sex×activity state had a significant effect on the postprandial body surface temperature (*F*=11.915, *P*=0.0035, Table 2B and Fig. 2B). The body temperature differed significantly between the sexes only in the rested cohort (Tukey HSD test: difference in emmeans=1.2, *P*=0.0001) and not in the active cohort (difference in emmeans=0.3, *P*=0.642).

## DISCUSSION

Fruit bats show remarkable adaptation to high-sugar diets compared to insectivorous bats (Meng et al., 2016). One key adaptation is their ability to metabolise exogenous dietary sugars and fuel active flight (Amitai et al., 2010). But the sex-differences in postprandial metabolic traits in fruit bats are poorly understood. Here, we addressed an open question: whether metabolic traits are expressed differently in each sex of the fruit-eating bat, *Cynopterus sphinx*. We found that female bats have slightly higher blood glucose and body surface temperature in their postprandial state, compared to males. Notably, the level of sex differences in both traits measured from rested bats was only significant at specific sampling times of the assay. Strikingly, the sex differences in both traits eroded with flight. These findings elucidate that while sex differences in metabolic traits can be detected, the outcomes are significantly affected by flight in *C. sphinx*.

Earlier studies on several species of frugivore and nectarivore bats report unusually high postprandial blood glucose levels for their body size. For instance, the average postprandial blood glucose level (irrespective of sex) is around 8 mmol/l in the fruit bat, *Epomophorus wahlbergi* (body mass ∼100 ***g***) (Mqokeli and Downs, 2012); around 15 mmol/l in the nectar bat, *Eonycteris spelaea* (body mass ∼60 ***g***) (Peng et al., 2017); and around 25 mmol/l in the nectar bat, *Glossophaga soricina* (body mass ∼10 ***g***) (Kelm et al., 2011). Notably, our results support previous findings that *C. sphinx* has high blood glucose for their body mass regardless of sex (>30 mmol/l here and ∼24 mmol/l in Peng et al., 2017). When compared to a similar-sized bat *E. spelaea* within the same study, *C. sphinx* was found to have blood glucose almost 10 mmol/l higher (Peng et al., 2017). Moreover, *C. sphinx* has postprandial blood glucose more than 20 mmol/l compared to the commonly used laboratory rats (Omer et al., 2004). Whether such high blood glucose levels could negatively affect the bats’ health remains unclear.

We note that the peak postprandial blood glucose values in *C. sphinx* reported in our study and the earlier study (Peng et al., 2017) are markedly different from the values sampled from individuals in their natural habitat. The peak postprandial blood glucose that we recorded from foraging bats in the wild was 8.83±0.55 mmol/l in males (*n*=16) and 9.36±0.60 mmol/l in females (*n*=7), respectively (unpublished). Although the blood glucose level measured in wild-caught bats might be confounded by physical activity and the amount of sugar consumed in the bats’ natural diet, the level is noticeably lower than the values recorded in the lab. Thus, the high blood glucose levels reported from studies on fruit bats kept in captivity should be interpreted with caution.

Previous studies that investigated sex-general postprandial blood glucose in fruit and nectar bats report temporal variation in the trait: reduction in blood glucose level over time in individuals kept at rest during the assays (Kelm et al., 2011; Meng et al., 2016; Peng et al., 2017). In our study, the change in postprandial blood glucose level over time was identical in both male and female bats. Blood glucose values recorded at 100 min were similar to the preprandial level in both sexes. Thus, in the absence of flight activity, it takes 100 min for both male and female bats to lower their postprandial blood glucose to fasting level. So, there are mechanisms that help fruit-eating bats to regulate glucose metabolism while resting. Higher levels of carbohydrate metabolising enzymes (e.g. disaccharidase, amylase, maltase and sucrase) secreted in the gut of fruit bats could be one plausible mechanism that helps regulate blood glucose level in the absence of activity (Hernandez and Martinez del Rio, 1992; Ogunbiyi and Okon, 1976).

The thermal imaging method allowed us to detect body temperature changes in the bats to the nearest 0.1°C. We found three intriguing pieces of evidence that adds to our understanding of glucose metabolism in fruit bats. Firstly, the immediate difference in body surface temperature between the pre- and postprandial states provides support for employing skin temperature differences to detect discrete metabolic changes in these small mammals. The data also support the notion that fruit bats release heat during digestion of sugars like other homeotherms (Stock, 1999). Secondly, female bats have slightly higher body temperature than males in their post-absorptive state. The fact that the increase in body temperature is directly related to glucose metabolism implies that female bats might have evolved a higher rate of metabolism than males. And lastly, our findings indicate that male and female individuals of the same species could regulate body temperature differently during metabolising dietary sugars.

Our results confirm that flight exert significant effects on postprandial metabolism in fruit bats. Similar to findings reported in earlier studies (Kelm et al., 2011; Peng et al., 2017), we found that the rested bats show higher postprandial blood glucose level than active bats. Meaning that fruit bats use dietary sugar to fuel their flight. We observed a different pattern in body surface temperature: where rested individuals showed lower temperature values than active bats. An interesting addition to the existing literature is that flight affects blood glucose level and body surface temperature equally in both sexes. We predict that because flight is an expensive and essential activity for both sexes, it would be less likely to detect sex differences in metabolic traits sampled from active bats.

Although only slightly different from males, female bats have generally higher levels of postprandial metabolism. Morphological differences between the sexes provide no clear explanation for the sex differences found in the metabolic traits because the mean body mass of females [mean=47.84 (s.d.=4.2)] is only marginally higher than male bats [mean=45.16 (s.d.=5.7)] used in our study. Sex difference in pancreas size and insulin level could explain the observed metabolic differences, but empirical support is poor. Confirming the difference in expression levels of a glucose transporter *SLC2A2* gene and its protein product Glut2 in each sex of *C. sphinx* will add another explanation for observed sex differences in metabolism (Meng et al., 2016). Nonetheless, our findings provide new insights to sex differences found in the general ecology of *C. sphinx*. Female bats disperse farther from their natal grounds and forage over larger home range area than males (Gopukumar et al., 2005; Marimuthu et al., 1998). Based on our findings, we contend that the higher rate of glucose metabolism provides higher energy reserves to sustain physical activity in female *C. sphinx*. Evidence indicates that the fruit bat *Rousettus aegyptiacus* exhibits sex-specific foraging behaviours (Barclay and Jacobs, 2011; Harten et al., 2018). It is, therefore, plausible that sex differences in metabolic traits might be commonly observed in frugivorous bats. Moreover, the finding that sex differences in metabolic traits are affected by flight is novel and invokes more general questions regarding sex-specific life-history evolution in bats. Whether metabolic and behavioural differences affect underpinning physiological traits and whether such effects resonate across life-history and confer sex differences in longevity outcomes are questions for future studies to explore.

### Conclusion

Our findings support the predictions: the metabolic trait values are generally higher in females compared to males, and that the sex differences in metabolic traits are affected by flight in the fruit bats, *C. sphinx*. The level of postprandial blood glucose and body surface temperature significantly differed between the sexes only when bats were kept at rest, and not when allowed to fly during the assay. Two inferences can be made from these results: (1) sex differences in metabolic traits are detectable in the absence of flight, and (2) flight is an expensive form of locomotion that it affects postprandial metabolic homeostasis equally in both sexes. More generally, our study elucidates the context-dependency of sex-differences in the metabolic traits: time of the assay and flight activity. Future studies should thus replicate their assays across a temporal scale rather than one-time sampling, and different activity states to detect signals of sex specificity in the traits. Finally, our findings have general implications for future studies that investigate similar phenotypes in other groups of bats: the results can be sex specific, and important information may be lost if the sex of the individual is not considered.

## MATERIALS AND METHODS

### Ethics statement

This study was conducted with ethics permission obtained from the Institutional Ethics Committee of Madurai Kamaraj University (Tamil Nadu, India). Bats were kept in captivity only for the duration of the study and were immediately released into their roosting sites from where they were originally caught. Bats were fed with 40% sugar solution before releasing to ensure that the individuals were not fatigued. No bats were sacrificed for this study.

### Sampling of bats

In total, 60 bats (*n*_males_=30, *n*_females_=30) were used in this study. The average body mass of male and female bats was 45.16 ***g*** (s.d.=5.7), and 47.84 ***g*** (s.d.=4.2) respectively. Bats were captured from known foraging and roosting sites around Palayamkottai town (Tamil Nadu, India). The local habitat in the sampling sites is a semi-urban environment, where bats roost in drooping fronds of palmyra palm trees and feed on fruiting trees in the area. We sampled bats in non-breeding seasons (in May, June, August, and September) across three years, 2014 to 2016. For capture, we used mist nets (4 m×2 m, 38 mm total mesh size polyester nets, Avinet, USA) or custom-built hoop nets, attached to extendable aluminium poles. The age-class of the captured individuals were immediately determined by examining the extent of wearing in canine teeth (Brunet-Rossinni and Wilkinson, 2009). Only adult bats were retained for the study. Retained bats were translocated into an animal house in St. John's College (Palayamkottai, Tamil Nadu, India). The animal house was equipped with several custom-built enclosures (each measuring 1 m×0.5 m×1 m) that were placed within rooms maintained at natural ambient temperature, humidity and light cycle. Each enclosure housed four same-sexed bats that had access to *ad libitum* fresh food (freshly cut fruits placed in a sterile plastic tray) and water (with no added sugar). Fresh food and water were replenished every 12 h in each enclosure.

### Study design

We first assessed temporal changes in postprandial levels of (a) blood glucose and (b) body surface temperature in individuals kept at rest. Then, we measured postprandial levels of (c) blood glucose and (d) body surface temperature across rested and active individuals. Each of the four assays was performed several months apart, with different male and female individuals used in each assay. All assays were performed between 19h00 and 21h00, which corresponds to the bats’ natural feeding time (Marimuthu et al., 1998). Individuals fasted for 10 h before the assays. At the time of the assay, we handfed the individuals with 1.5 ml standard liquid diet (hereafter, ‘standard diet’) with a sterile feeding syringe. The standard diet contained 0.089 g/ml of sugar along with trace amounts of minerals and vitamin. We followed the same feeding method in all four assays. Our approach of controlled feeding allowed us to standardise the amount of sugar consumed by each individual (irrespective of their sex, body mass and satiety level), and minimise any confounding effects attributable to inter-individual feeding differences.

### Data collection

We measured body mass (***g***) and forearm length (mm) of each individual immediately prior to the assays. Both pre- and postprandial blood glucose levels were measured using an instant glucometer (OneTouch^®^ SelectSimple™ with detection range=1.1 to 33.3 mmol/l of blood glucose). We used 5 µl of blood drawn from the uropatagium area (between the tail and tibia) of the animal using a sterile needle (6 mm needle, Becton-Dickinson) for instant glucometer measurements. Nonetheless, there were instances when the glucometer was unable to detect blood glucose levels because of values outside its detection range. Particularly, the postprandial glucose level at 20 min (see below in blood glucose assay a) and at 30 min (in assay c) in resting bats were difficult to measure with the glucometer. In such cases, 100 µl of blood was sampled from the uropatagium area of the individual and glucose level was immediately assessed on a blood analyser (Chem 7, ERBA Manheim GmBH).

A handheld thermal imaging camera (Model E4, FLIR Systems Inc., USA; detection range -20° to 250°C) was used to measure body surface temperature from individual bats. Thermal imaging of bats was performed inside a room maintained at 28°C to minimise the effect of ambient temperature. Images were retrieved from the camera and manually corrected for background noise (infrared reflectance from the surrounding surface) in the FLIR tools software (Tattersall, 2016). We then estimated the mean body surface temperature of each bat from values taken across three data points (chest, upper and lower abdomen) drawn on the anterior part of the animal, in the thermal image in FLIR tools software (version 5.x, FLIR Systems Inc.). It is important to note that the anatomical region of the animal affects body surface temperature measurements (McCafferty et al., 2015; Taylor et al., 2014). Temperature measured from the wing area has been particularly employed in studies investigating thermoregulation in bats. Bats living in the tropics use wings as outlets to lose or preserve heat during flying in warm or cold conditions (Reichard et al., 2010). So, we avoided wings and measured body surface temperature from the anterior part of the animal to detect temperature changes associated with postprandial metabolism. The non-invasive sampling of body surface temperature allowed us to use a slightly higher sample size (*n*=30), compared to the invasive sampling of blood glucose (*n*=24).

#### (a) Assaying change in postprandial blood glucose in resting bats over time

Eight bats (*n*_males_=4 and *n*_females_=4) were used in this assay. Preprandial level of blood glucose (denoted as 0 min reading in the figures) was first measured. After 30 min from measuring the preprandial blood glucose, each bat was hand-fed with 1.5 ml of standard diet and retained in individual clean cloth bags. After the one-time feeding, postprandial blood glucose level of the individuals was measured at 20 min intervals, for a total of 100 min.

#### (b) Assaying change in postprandial body surface temperature in resting bats over time

Ten bats (*n*_males_=5, *n*_females_=5) were used in this assay. Similar to the blood glucose assay (a), body surface temperature was recorded in pre- and postprandial states of the bats. Once fed with the standard diet, thermal images of the individuals were taken every 15-min, completing the assay at 60 min.

#### (c) Postprandial blood glucose in rested and active bats

We first measured postprandial blood glucose level from individuals (*n*_males_=4, *n*_females_=4) that were kept at rest. In the rest group, individuals were fed with the standard diet and held at complete rest for 30 min in separate clean cloth bags. We measured the postprandial blood glucose using the blood analyser. Then, with a separate group of bats (*n*_males_=4, *n*_females_=4), we measured the postprandial blood glucose values from active individuals. In the active group, 10 min after feeding, each bat was allowed to fly for 5 min within a cubicle (2.5 m×2 m×2 m). Bats could only fly inside the cubicle with no means to rest. We monitored their activity within the cubicle and disturbed the individual if it was found to rest on the floor. Thus, we ensured that bats were active throughout the 5 min spent inside the cubicle. Only one bat was introduced into the cubicle at a given time. The individual was removed from the cubicle and allowed to rest for 5 min in a cloth bag. We then measured the postprandial blood glucose using the instant glucometer. In both groups, postprandial blood glucose was only measured once from each individual.

#### (d) Postprandial body surface temperature in rested and active bats

We followed the same method described in assay (c) to measure postprandial body surface temperature in bats that were either kept at rest or allowed to fly. We first recorded thermal images of individuals (*n*_males_=5, *n*_females_=5) that were kept at rest for 30 min, immediately after feeding. Then, with a separate group of bats (*n*_males_=5, *n*_females_=5), we recorded thermal images of individuals that were allowed to fly for 5 min after feeding.

### Data analyses

All analyses were performed in the R statistical environment v3.6 (R Development Core Team, 2020). Graphical plots were made using the GraphPad Prism software (v8.1.0) and ggplot2 package (Wickham, 2016) in R.

#### (i) Linear mixed-effect analyses for estimating sex-differences in postprandial blood glucose and body surface temperature in rested bats

The data from assays measuring postprandial levels of (a) blood glucose and (b) body surface temperature over time in rested bats were analysed using linear mixed-effect (lmer) statistical models in R. Histogram of the raw data indicated that the blood glucose data is left-skewed and body surface temperature is normally distributed. We log-transformed the blood glucose data to adjust for left skewness and used the transformed data in the lmer model, assuming Gaussian distribution.

Lmer models for blood glucose and body surface temperature data were analysed separately. In each of these two models, we included the respective trait value as the response variable, along with sex (two levels), time of the assay (five or six levels depending on the trait) and the two-way interaction between the two factors (sex×time of the assay) as fixed effects. Body size index (ratio of body mass and forearm length) of the individuals was included as a fixed covariate in each model. Furthermore, to account for repeated sampling of the traits from the same individual over time, we included ‘individual ID’ as a random factor in both models to account for compounding effects. Thus, a global model was built for each trait with fixed effects and first-order interaction between the fixed effects, the covariate and random effect terms.

We then performed a model reduction analysis using *dredge* function in MuMIn package in R (Barton and Barton, 2019) to determine the best-fit model for each response variable. The dredge function performed an automated stepwise elimination of each fixed effect term in the model. It provided the corrected Akaike Information Criterion (AICc) values for the global model and each of the reduced models. We restricted model selection to the top six models. From the chosen six models, we calculated the relative variable importance (RVI) for each fixed effect term. RVI indicates the likelihood that a particular fixed effect term would be present among the six best-fit models. RVI for each fixed effect was calculated as the sum of the weight of models in which the fixed effect term was retained (Burnham and Anderson, 2002). We observed that the best fit model (based on the AICc score) was not informative and that using the reduced model for further analyses undermined our efforts to detect sex differences in the traits while accounting for effects of sampling time. So, we proceeded with the global model for further analyses. Results for the six best-fit models from the dredge analysis are provided in Tables S1 and S2.

Parameter estimates for the fixed effects in the global models were estimated using the maximum likelihood method in the lme4 package (Bates et al., 2015). *P*-value significance and Chi-squared values for each fixed effect were estimated using the Wald's III Chi-squared test in car package (Fox and Weisberg, 2011). The standard deviation (s.d.) in response variable attributable to the random effect was estimated using the restricted maximum likelihood method in lme4 package. *P*-value significance for the random effect term was determined using *ranova* function in lmerTest package (Kuznetsova et al., 2017).

We then derived estimated marginal means (emmeans that has no units) and±1 standard error of both traits for the interaction term ‘sex×time of the assay’, using the *emmeans* package in R (Searle et al., 1980). Emmeans allowed us to correct the trait means for confounding effects attributable to the body size of individuals and repeated measurements made on the same individual over time. We used emmeans values for making graphical plots. Finally, we analysed for significance in differences in trait means (mean value estimated from raw data) across all pairwise combinations of sex and time of the assay. For this pairwise comparison analysis, we used a posthoc analytical framework using the TukeyHSD test in R (results not shown in Tables 1A and B).

#### (ii) Analysing the effect of flight on the level of sex-differences in each trait

We analysed the effect of flight on the level of sex differences in both traits measured in assays c and d using linear models. Since the individuals were only sampled once in these assays involving rested and active individuals, we omitted the term ‘individual ID’ in the models. Separate linear models were built for each trait. The models included the respective trait value as the response variable; sex (two levels), activity state (two levels) and the interaction between sex and activity-state as factors, and body size index as the covariate. Parameter estimates and *P*-value significance for each factor in the model were estimated using ANOVA test in R. We then estimated the emmeans±standard error of each trait for the interaction term ‘sex×activity-state’ that adjusted the response variable for effects of body size. We used the raw data of both response variables for calculating emmeans. Furthermore, TukeyHSD test was used to determine the significant differences in trait means across all pairwise combinations of sex and activity states.

## Supplementary Material

Supplementary information

## References

[BIO053926C1] Ahn, M., Anderson, D. E., Zhang, Q., Tan, C. W., Lim, B. L., Luko, K., Wen, M., Chia, W. N., Mani, S., Wang, L. C. et al. (2019). Dampened NLRP3-mediated inflammation in bats and implications for a special viral reservoir host. *Nat. Microbiol.* 4, 789-799. 10.1038/s41564-019-0371-3 30804542 PMC7096966

[BIO053926C2] Amitai, O., Holtze, S., Barkan, S., Amichai, E., Korine, C., Pinshow, B. and Voigt, C. C. (2010). Fruit bats (Pteropodidae) fuel their metabolism rapidly and directly with exogenous sugars. *J. Exp. Biol.* 213, 2693-2699. 10.1242/jeb.043505 20639431

[BIO053926C3] Austad, S. N. and Fischer, K. E. (2016). Sex differences in lifespan. *Cell Metab.* 23, 1022-1033. 10.1016/j.cmet.2016.05.019 27304504 PMC4932837

[BIO053926C4] Barclay, R. M. R. and Jacobs, D. S. (2011). Differences in the foraging behaviour of male and female Egyptian fruit bats (Rousettus aegyptiacus). *Can. J. Zool. Rev. Can. Zool.* 89, 466-473. 10.1139/z11-013

[BIO053926C5] Barton, K. and Barton, M. K. (2019). Package ‘MuMIn’. R package version 1.6 See https://cran.r-project.org/package=MuMIn.

[BIO053926C6] Bates, D., Mächler, M., Bolker, B. M. and Walker, S. C. (2015). Fitting linear mixed-effects models using lme4. *J. Stat. Softw.* 67, 1-48. 10.18637/jss.v067.i01

[BIO053926C7] Bonduriansky, R. and Chenoweth, S. F. (2009). Intralocus sexual conflict. *Trends Ecol. Evol.* 24, 280-288. 10.1016/j.tree.2008.12.005 19307043

[BIO053926C8] Brunet-Rossinni, A. K. and Wilkinson, G. (2009). Methods for age estimation and the study of senescence in bats. In *Ecological and Behavioral Methods for the Study of Bats* (eds T.H. Kunz and S. Parsons), pp.315-325. Johns Hopkins University Press.

[BIO053926C9] Bruno, A., Biller, J., Adams, H. P., Jr, Clarke, W. R., Woolson, R. F., Williams, L. S. and Hansen, M. D. (1999). Acute blood glucose level and outcome from ischemic stroke. Trial of ORG 10172 in Acute Stroke Treatment (TOAST) Investigators. *Neurology* 52, 280-284. 10.1212/WNL.52.2.280 9932944

[BIO053926C10] Burnham, K. P. and Anderson, D. R. (2002). *Model Selection and Multimodel Inference: A Practical Information-Theoretic Approach*. New York: Springer Science+Business media.

[BIO053926C11] Ceriello, A. (2003). The possible role of postprandial hyperglycaemia in the pathogenesis of diabetic complications. *Diabetologia* 46, M9-M16. 10.1007/s00125-002-0931-5 12652353

[BIO053926C12] Clancy, D. J. (2008). Variation in mitochondrial genotype has substantial lifespan effects which may be modulated by nuclear background. *Aging Cell* 7, 795-804. 10.1111/j.1474-9726.2008.00428.x 18727704

[BIO053926C13] Clutton-Brock, T. H. and Isvaran, K. (2007). Sex differences in ageing in natural populations of vertebrates. *Proc. R. Soc. B Biol. Sci.* 274, 3097-3104. 10.1098/rspb.2007.1138 PMC2293943 17939988

[BIO053926C14] Fox, J. and Weisberg, S. (2011). *An R Companion to Applied Regression*, 2nd edn. Sage.

[BIO053926C15] Gopukumar, N., Karuppudurai, I. and Doss, D. P. S. (2005). Dispersal patterns of the short-nosed fruit bat Cynopterus sphinx (Chiroptera: Pteropodidae). *Mamm. Biol.* 70, 122-125. 10.1016/j.mambio.2004.10.001

[BIO053926C16] Harrison, J. F. and Roberts, S. P. (2000). Flight respiration and energetics. *Annu. Rev. Physiol.* 62, 179-205. 10.1146/annurev.physiol.62.1.179 10845089

[BIO053926C17] Harten, L., Matalon, Y., Galli, N., Navon, H., Dor, R. and Yovel, Y. (2018). Persistent producer-scrounger relationships in bats. *Sci. Adv.* 4, e1603293 10.1126/sciadv.1603293 29441356 PMC5810609

[BIO053926C18] Hernandez, A. and Martinez del Rio, C. (1992). Intestinal disaccharides in five species of phyllostomoid bats. *Comp. Biochem. Physiol. B Comp. Biochem.* 103, 105-111. 10.1016/0305-0491(92)90420-V 1451428

[BIO053926C19] Huang, X., Hancock, D. P., Gosby, A. K., McMahon, A. C., Solon, S. M. C., Le Couteur, D. G., Conigrave, A. D., Raubenheimer, D. and Simpson, S. J. (2013). Effects of dietary protein to carbohydrate balance on energy intake, fat storage, and heat production in mice. *Obesity* 21, 85-92. 10.1002/oby.20007 23404943

[BIO053926C20] Karp, N. A., Mason, J., Beaudet, A. L., Benjamini, Y., Bower, L., Braun, R. E., Brown, S. D. M., Chesler, E. J., Dickinson, M. E., Flenniken, A. M. et al. (2017). Prevalence of sexual dimorphism in mammalian phenotypic traits. *Nat. Commun.* 8, 15475 10.1038/ncomms15475 28650954 PMC5490203

[BIO053926C21] Kelm, D. H., Simon, R., Kuhlow, D., Voigt, C. C. and Ristow, M. (2011). High activity enables life on a high-sugar diet: blood glucose regulation in nectar-feeding bats. *Proc. Biol. Sci.* 278, 3490-3496. 10.1098/rspb.2011.0465 21490011 PMC3189374

[BIO053926C22] Kuznetsova, A., Brockhoff, P. B. and Christensen, R. H. B. (2017). lmerTest package: tests in linear mixed effects models. *J. Stat. Softw.* 82, 1-26. 10.18637/jss.v082.i13

[BIO053926C23] Marimuthu, G., Rajan, K. E., Koilraj, A. J., Isaac, S. S. and Balasingh, J. (1998). Observations on the foraging behavior of a tent roosting megachiropteran bat Cynopterus sphinx. *Biotropica* 30, 321-324. 10.1111/j.1744-7429.1998.tb00066.x

[BIO053926C24] Mauvais-Jarvis, F. (2015). Sex differences in metabolic homeostasis, diabetes, and obesity. *Biol. Sex. Differ.* 6, 14 10.1186/s13293-015-0033-y 26339468 PMC4559072

[BIO053926C25] McCafferty, D. J., Gallon, S. and Nord, A. (2015). Challenges of measuring body temperatures of free-ranging birds and mammals. *Anim. Biotelemet.* 3, 33 10.1186/s40317-015-0075-2

[BIO053926C26] Meng, F., Zhu, L., Huang, W., Irwin, D. M. and Zhang, S. (2016). Bats: Body mass index, forearm mass index, blood glucose levels and SLC2A2 genes for diabetes. *Sci. Rep.* 6, 29960 10.1038/srep29960 27439361 PMC4954980

[BIO053926C27] Mqokeli, B. R. and Downs, C. T. (2012). Blood plasma glucose regulation in Wahlberg's epauletted fruit bat. *Afr. Zool.* 47, 348-352. 10.3377/004.047.0218

[BIO053926C28] Munshi-South, J. and Wilkinson, G. S. (2010). Bats and birds: exceptional longevity despite high metabolic rates. *Ageing Res. Rev.* 9, 12-19. 10.1016/j.arr.2009.07.006 19643206

[BIO053926C29] Nagarajan-Radha, V., Aitkenhead, I., Clancy, D. J., Chown, S. L. and Dowling, D. K. (2020). Sex-specific effects of mitochondrial haplotype on metabolic rate in Drosophila melanogaster support predictions of the Mother's Curse hypothesis. *Philos. Trans. R. Soc. Lond. B Biol. Sci.* 375, 20190178 10.1098/rstb.2019.0178 31787038 PMC6939370

[BIO053926C30] Nagashima, K., Nakai, S., Matsue, K., Konishi, M., Tanaka, M. and Kanosue, K. (2003). Effects of fasting on thermoregulatory processes and the daily oscillations in rats. *Am. J. Physiol. Regul. Integr. Comp. Physiol.* 284, R1486-R1493. 10.1152/ajpregu.00515.2002 12736180

[BIO053926C31] Norheim, F., Hasin-Brumshtein, Y., Vergnes, L., Chella Krishnan, K., Pan, C., Seldin, M. M., Hui, S. T., Mehrabian, M., Zhou, Z., Gupta, S. et al. (2019). Gene-by-sex interactions in mitochondrial functions and cardio-metabolic traits. *Cell Metab.* 29, 932-949.e4. 10.1016/j.cmet.2018.12.013 30639359 PMC6447452

[BIO053926C32] Ogunbiyi, O. A. and Okon, E. E. (1976). Studies on the digestive enzymes of the African fruit bat Eidolon helvum (Kerr). *Comp. Biochem. Physiol. A Comp. Physiol.* 55, 359-361. 10.1016/0300-9629(76)90061-X 9251

[BIO053926C33] O'Mara, M. T., Wikelski, M., Voigt, C. C., Ter Maat, A., Pollock, H. S., Burness, G., Desantis, L. M. and Dechmann, D. K. (2017). Cyclic bouts of extreme bradycardia counteract the high metabolism of frugivorous bats. *eLife* 6, e26686 10.7554/eLife.26686 PMC5605195 28923167

[BIO053926C34] Omer, A., Duvivier-Kali, V. F., Aschenbach, W., Tchipashvili, V., Goodyear, L. J. and Weir, G. C. (2004). Exercise induces hypoglycemia in rats with islet transplantation. *Diabetes* 53, 360-365. 10.2337/diabetes.53.2.360 14747286

[BIO053926C35] Partridge, L., Gems, D. and Withers, D. J. (2005). Sex and death: what is the connection? *Cell* 120, 461-472. 10.1016/j.cell.2005.01.026 15734679

[BIO053926C36] Peng, X. W., He, X. Y., Liu, Q., Sun, Y. X., Liu, H., Zhang, Q., Liang, J., Peng, Z., Liu, Z. X. and Zhang, L. B. (2017). Flight is the key to postprandial blood glucose balance in the fruit bats Eonycteris spelaea and Cynopterus sphinx. *Ecol. Evol.* 7, 8804-8811. 10.1002/ece3.3416 29152179 PMC5677482

[BIO053926C37] R Core Team (2020). R: A language and environment for statistical computing. R Foundation for Statistical Computing, Vienna, Austria. https://www.R-project.org/

[BIO053926C38] Reichard, J. D., Prajapati, S. I., Austad, S. N., Keller, C. and Kunz, T. H. (2010). Thermal windows on Brazilian free-tailed bats facilitate thermoregulation during prolonged flight. *Integr. Comp. Biol.* 50, 358-370. 10.1093/icb/icq033 20811514 PMC2931312

[BIO053926C39] Searle, S. R., Speed, F. M. and Milliken, G. A. (1980). Population marginal means in the linear-model - an alternative to least-squares means. *Am. Stat.* 34, 216-221. 10.1080/00031305.1980.10483031

[BIO053926C40] Stock, M. J. (1999). Gluttony and thermogenesis revisited. *Int. J. Obes. Relat. Metab. Disord.* 23, 1105-1117. 10.1038/sj.ijo.0801108 10578199

[BIO053926C41] Storz, J. F. and Kunz, T. H. (1999). Cynopterus sphinx. *Mamm. Species* 613, 1-8. 10.2307/3504423

[BIO053926C42] Tarka, M., Guenther, A., Niemelä, P. T., Nakagawa, S. and Noble, D. W. A. (2018). Sex differences in life history, behavior, and physiology along a slow-fast continuum: a meta-analysis. *Behav. Ecol. Sociobiol.* 72, 132 10.1007/s00265-018-2534-2 30100667 PMC6060830

[BIO053926C43] Tattersall, G. J. (2016). Infrared thermography: a non-invasive window into thermal physiology. *Comp. Biochem. Physiol. A Mol. Integr. Physiol.* 202, 78-98. 10.1016/j.cbpa.2016.02.022 26945597

[BIO053926C44] Taylor, N. A. S., Tipton, M. J. and Kenny, G. P. (2014). Considerations for the measurement of core, skin and mean body temperatures. *J. Therm. Biol.* 46, 72-101. 10.1016/j.jtherbio.2014.10.006 25455943

[BIO053926C45] Thomas, S. P. (1975). Metabolism during flight in two species of bats, Phyllostomus hastatus and Pteropus gouldii. *J. Exp. Biol.* 63, 273-293. 1159367 10.1242/jeb.63.1.273

[BIO053926C46] Tower, J. (2017). Sex-specific gene expression and life span regulation. *Trends Endocrinol. Metab.* 28, 735-747. 10.1016/j.tem.2017.07.002 28780002 PMC5667568

[BIO053926C47] Turbill, C., Law, B. S. and Geiser, F. (2003). Summer torpor in a free-ranging bat from subtropical Australia. *J. Therm. Biol.* 28, 223-226. 10.1016/S0306-4565(02)00067-0

[BIO053926C48] Umminger, B. L. (1975). Body size and whole blood sugar concentrations in mammals. *Comp. Biochem. Physiol. A Comp. Physiol.* 52, 455-458. 10.1016/S0300-9629(75)80065-X 241543

[BIO053926C49] van Doorn, G. S. (2009). Intralocus sexual conflict. *Ann. N. Y. Acad. Sci.* 1168, 52-71. 10.1111/j.1749-6632.2009.04573.x 19566703

[BIO053926C50] Voigt, C. C. and Speakman, J. R. (2007). Nectar-feeding bats fuel their high metabolism directly with exogenous carbohydrates. *Funct. Ecol.* 21, 913-921. 10.1111/j.1365-2435.2007.01321.x

[BIO053926C51] Voigt, C. C. and Winter, Y. (1999). Energetic cost of hovering flight in nectar-feeding bats (Phyllostomidae: Glossophaginae) and its scaling in moths, birds and bats. *J. Comp. Physiol. B Bioch. Syst. Environ. Physiol.* 169, 38-48. 10.1007/s003600050191 10093905

[BIO053926C52] Welch, K. C., Herrera, M. L. G. and Suarez, R. K. (2008). Dietary sugar as a direct fuel for flight in the nectarivorous bat Glossophaga soricina. *J. Exp. Biol.* 211, 310-316. 10.1242/jeb.012252 18203985

[BIO053926C53] Wickham H. (2016). ggplot2: Elegant Graphics for Data Analysis. Springer-Verlag New York. ISBN 978-3-319-24277-4, https://ggplot2.tidyverse.org

[BIO053926C54] Winter, Y. and von Helversen, O. (1998). The energy cost of flight: do small bats fly more cheaply than birds? *J. Comp. Physiol. B Biochem. Syst. Environ. Physiol.* 168, 105-111. 10.1007/s003600050126 9542147

