# Supplementary material for: Sex differences in postprandial blood glucose and body surface temperature are contingent on flight in the fruit bat, Cynopterus sphinx
Source: Biol Open. 2021 Feb 17;10(2):bio053926. doi: 10.1242/bio.053926 (PMC7903995; doi:10.1242/bio.053926)
Supplement: Supplementary information [file biolopen-10-053926-s1.pdf]

Supplementary information

Table S1: AICc values derived from model reduction analysis of the postprandial blood glucose data (assay (a)). We used *dredge* function in MuMIn package to deduce the best fit model for the data. The models were analysed using maximum likelihood method. The global model included the sex, time of the assay and the two-way interaction between the two factors as fixed effects, individual ID as a random effect and body size index as the fixed covariate. Results for the best six models are shown below.

Model rank	Sex	Time of the assay	Sex × time of the assay	Body size index	Df	logLik	AICc	Delta	Weight
1	-	+	-	-	8	-4.327	28.3	0	0.338
2	-	+	-	+	9	-2.842	28.4	0.07	0.325
3	-	+	+	-	9	-3.105	28.9	0.6	0.25
4	+	+	-	+	10	-2.624	31.2	2.85	0.081
5	+	+	+	-	14	1.926	36.9	8.53	0.005
6	+	+	+	+	15	2.907	39.2	10.84	0.001

Table S2: AICc values derived from model reduction analysis of postprandial body surface temperature data (assay (b)). We used the *dredge* function in MuMIn package to deduce the best fit model for the data. The global model included the sex, time of the assay and the interaction between the two factors as fixed effects, individual ID as a random effect and body size index as the fixed covariate. Results for the best six models are shown below.

Model rank	Sex	Time of the assay	Sex × time of the assay	Body size index	Df	logLik	AICc	Delta	Weight
1	+	+	-	-	8	-36.924	93.4	0	0.503
2	+	+	-	+	9	-36.218	94.9	1.58	0.229
3	+	+	+	-	12	-31.908	96.2	2.89	0.119
4	-	+	-	+	8	-38.455	96.4	3.06	0.109
5	+	+	+	+	13	-31.141	98.4	5.03	0.041
6	-	+	-	-	7	-49.735	116.1	22.78	0

Biology Open • Supplementary information

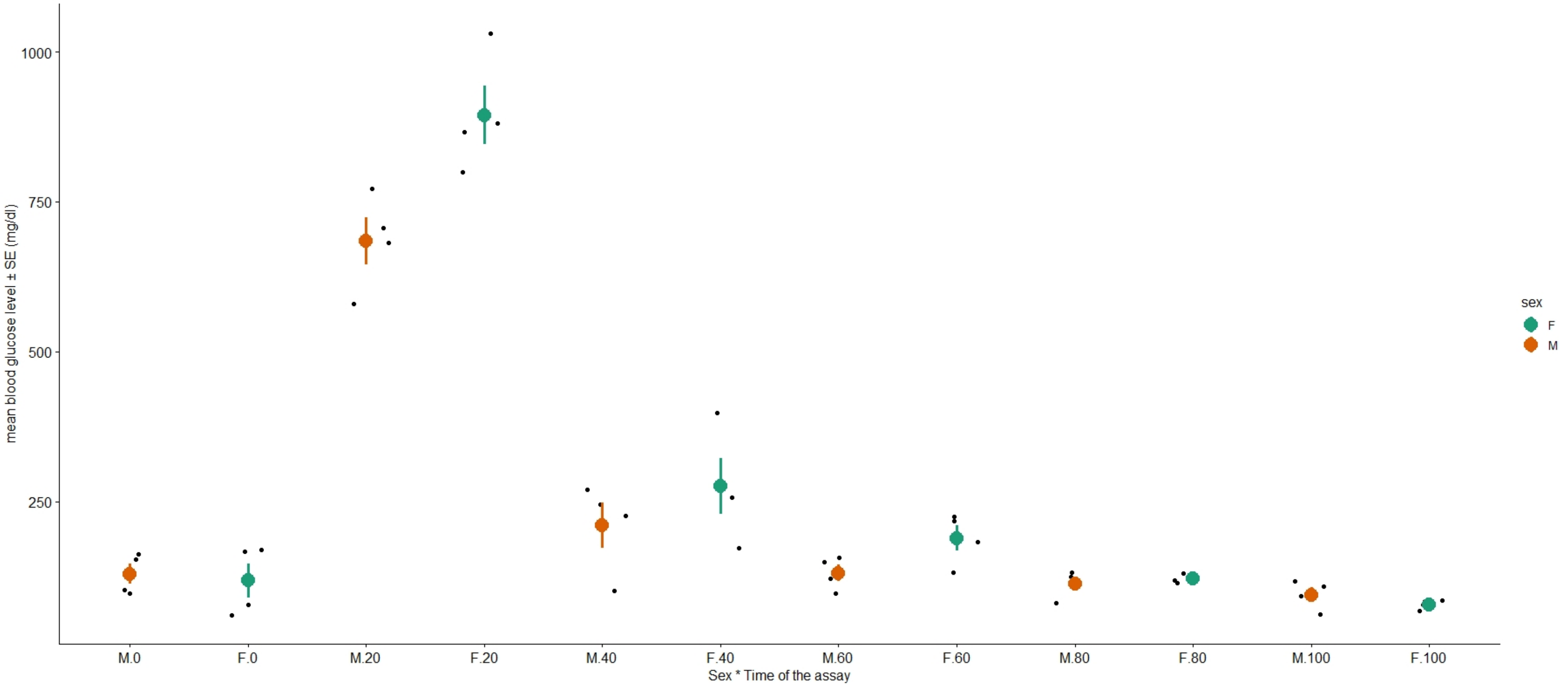

Fig S1. Mean (±SE) postprandial blood glucose (mg/dl) values estimated from raw data. The interaction between sex (male and female) and time of the assay (0 to 100 min) is shown in the horizontal axis. We used *ggpubr* package to draw this graph.

Biology Open • Supplementary information

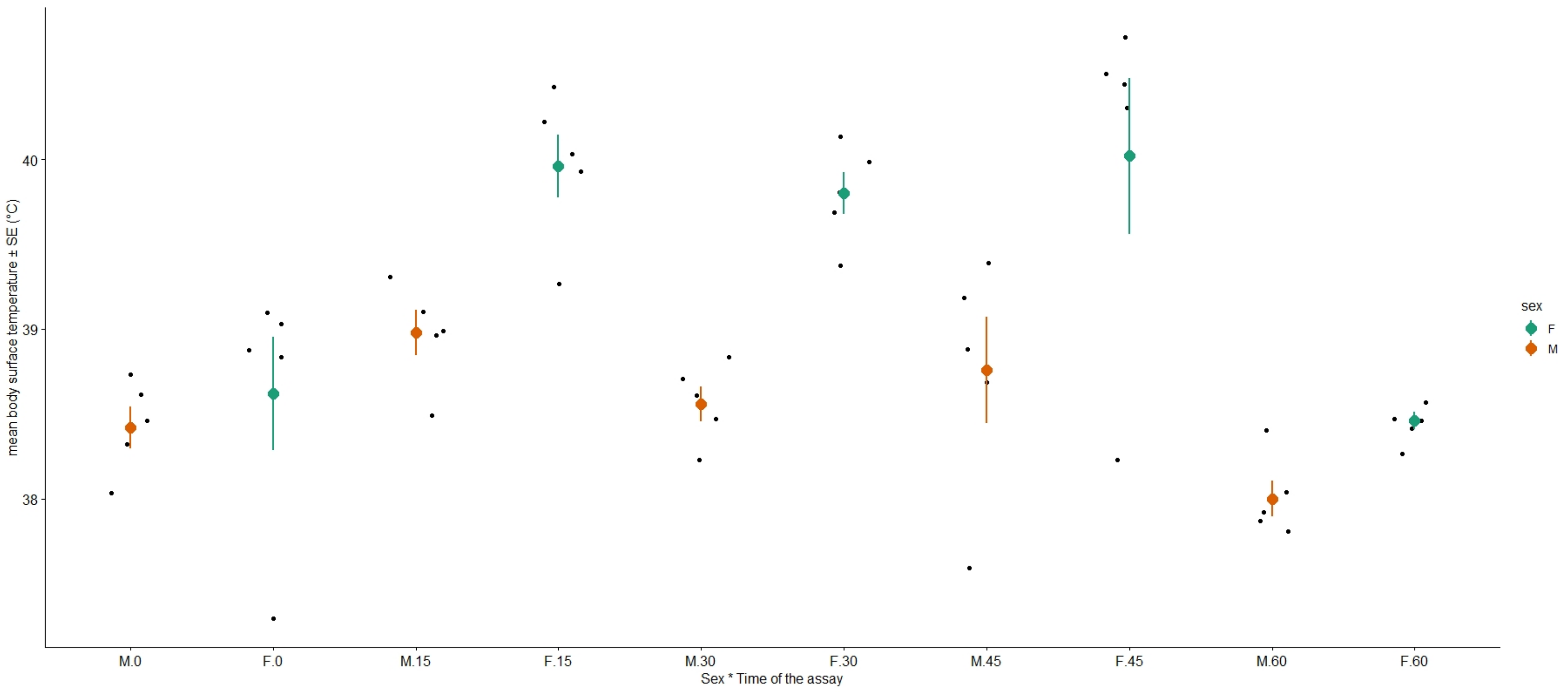

Fig S2. Mean (±SE) postprandial body surface temperature (°C) values estimated from raw data. The interaction between sex (male and female) and time of the assay (0 to 60 min) is shown in the horizontal axis.
